# Associations of serum short-chain fatty acids with circulating immune cells and serum biomarkers in patients with multiple sclerosis

**DOI:** 10.1038/s41598-021-84881-8

**Published:** 2021-03-04

**Authors:** Stephanie Trend, Jonatan Leffler, Anderson P. Jones, Lilian Cha, Shelley Gorman, David A. Brown, Samuel N. Breit, Allan G. Kermode, Martyn A. French, Natalie C. Ward, Prue H. Hart

**Affiliations:** 1 Telethon Kids Institute, University of Western Australia, PO Box 855, West Perth, WA 6872 Australia; 2 Centre for Neuromuscular and Neurological Disorders, Perron Institute for Neurological and Translational Science, University of Western Australia, Perth, WA Australia; 3 Institute for Clinical Pathology and Medical Research, NSW Health Pathology and Westmead Institute for Medical Research, Centre for Immunology and Allergy Research, Westmead, University of Sydney, Sydney, NSW Australia; 4 St Vincent’s Centre for Applied Medical Research, St Vincent’s Hospital, University of NSW, Sydney, NSW Australia; 5 Institute for Immunology and Infectious Disease, Murdoch University, Perth, WA Australia; 6 UWA Medical School and School of Biomedical Sciences, University of Western Australia, Perth, WA Australia; 7 Medical School, University of Western Australia, Perth, WA Australia; 8 School of Public Health, Curtin Health Innovation Research Institute, Curtin University, Perth, WA Australia

**Keywords:** Immunology, Adaptive immunity, Autoimmunity, Cytokines, Immunological disorders, Inflammation, Lymphocytes, Neuroimmunology

## Abstract

Altered composition of gut bacteria and changes to the production of their bioactive metabolites, the short-chain fatty acids (SCFAs), have been implicated in the development of multiple sclerosis (MS). However, the immunomodulatory actions of SCFAs and intermediaries in their ability to influence MS pathogenesis are uncertain. In this study, levels of serum SCFAs were correlated with immune cell abundance and phenotype as well as with other relevant serum factors in blood samples taken at first presentation of Clinically Isolated Syndrome (CIS; an early form of MS) or MS and compared to healthy controls. There was a small but significant reduction in propionate levels in the serum of patients with CIS or MS compared with healthy controls. The frequencies of circulating T follicular regulatory cells and T follicular helper cells were significantly positively correlated with serum levels of propionate. Levels of butyrate associated positively with frequencies of IL-10-producing B-cells and negatively with frequencies of class-switched memory B-cells. TNF production by polyclonally-activated B-cells correlated negatively with acetate levels. Levels of serum SCFAs associated with changes in circulating immune cells and biomarkers implicated in the development of MS.

## Introduction

Multiple sclerosis (MS) is a complex inflammatory neurological disorder of unknown aetiology involving abnormalities in a range of immune cells, including T-cells and B-cells. The variety of immune cells implicated in the immunopathogenesis of MS raises the possibility that alongside expression of genetic risk factors^1^, agents with immunomodulatory activity on immune cells may contribute to environmental risks for developing the disease.

The human microbiome is a major determinant of human health over the life course, providing a range of soluble factors that can contribute to regulation of the human immune system. Healthy people have a “core microbiome” composition which has been shown to be disturbed in several disease states. Studies investigating the gut microbiome in patients with MS have demonstrated evidence of dysbiosis (reviewed in^2,3^). However, outcomes have been variable between studies, with mostly non-replicable findings of changes in the abundance of diverse groups of bacteria^4–8^. Determining the clinical importance of changes in specific groups of microbes is therefore difficult to achieve. Examining downstream products of microbial metabolism might provide a species-independent assessment of the immune modulating activities of the gut microbiome. Recently, the composition of the gut microbiome was investigated alongside metabolomics, revealing decreased levels of the short-chain fatty acids (SCFAs) butyrate and propionate in the gut of MS patients^9^. In one study, the proportions of SCFA-producers *Roseburia*, *Coprococcus* and *Blautia* were reduced by between 2- and fivefold in patients with MS^10^. The proposal that the changes in gut microbiota in MS patients translate to changes in the SCFAs they produce have been supported by reports of lower serum levels of butyrate^10^ and propionate^11^ in the serum of MS patients.

The SCFAs acetate, propionate and butyrate, are the main metabolites produced in the human colon by bacterial anaerobic fermentation of indigestible polysaccharides such as dietary fibres and resistant starch; very small amounts of SCFAs are also produced by colonic microbiota from amino acid metabolism (reviewed in^12^). SCFAs are small organic monocarboxylic acids of up to six carbon atoms in length. These SCFAs may act locally in the gut, for example by maintaining the integrity of the intestinal epithelium, provision of energy for colonocytes and induction of regulatory cells^13,14^. However, SCFAs may also act in distant tissues. Levels of SCFAs in stool do not necessarily predict levels of SCFAs in serum and distant lymphoid tissues because of differences in the structural integrity of the gut epithelium and/or the expression of receptors that mediate transport of SCFA across the colonocytes (reviewed in^12^). An investigation of the circulating levels of SCFAs provides one mechanism to investigate the collective and indirect effects of the microbiome on distal immune cells.

Within the immune system, SCFAs are generally considered anti-inflammatory although their mechanisms of action require further definition^3,12,15^. By binding to G-protein coupled receptors on immune cells^16^, SCFAs may epigenetically regulate gene expression by inhibition of histone deacetylase activity and subsequent changes to lysines in nucleosomal histones^17–19^. SCFAs may also control lipid metabolism and gluconeogenesis in immune cells; the survival and activity of immune cells are determined in large part by flux through such metabolic pathways^20^.

In order to determine whether changes to levels of circulating SCFAs in patients with MS provide clues to drivers of their immune-mediated disease, levels of acetate, propionate and butyrate were measured in the serum of patients with clinically isolated syndrome (CIS) or MS and compared with the levels in sera from healthy controls. Of importance, the patients were therapy-naive and blood was collected as early as possible after their presentation of CIS or MS. We hypothesised that the putative immunoregulatory activities of the SCFAs would be associated with changes to the frequencies and functions of circulating immune cells including the ratios of effector to regulatory cells and other disease biomarkers in blood. The levels of the SCFAs were compared with the frequencies of, and cytokine production by, T- and B-cell subpopulations, and with levels of mRNA for tryptophan and arginine catabolic enzymes that regulate proliferation and metabolic activity of immune cells. The levels of the SCFAs were also compared with those of serum biomarkers that we, or others, have implicated in MS development and progression. These included IgG_3_^21,22^ and 25-hydroxy vitamin D (25(OH)D)^23^. In addition, serum levels of growth differentiation factor 15 (GDF15) were assessed as this factor has recently shown to be increased in patients with MS^24^. Frequently referred to as a stress responsive cytokine that increases during tissue injury and inflammation, the production by GDF15 by adipocytes, cardiomyocytes and macrophages may be related to the metabolic processes associated with SCFA function^25^. This study aimed to investigate the relationship between circulating SCFAs and MS development in our therapy-naive cohort.

## Results

### SCFA levels in the serum of patients with CIS or MS and association with clinical outcomes

**Figure 1:**
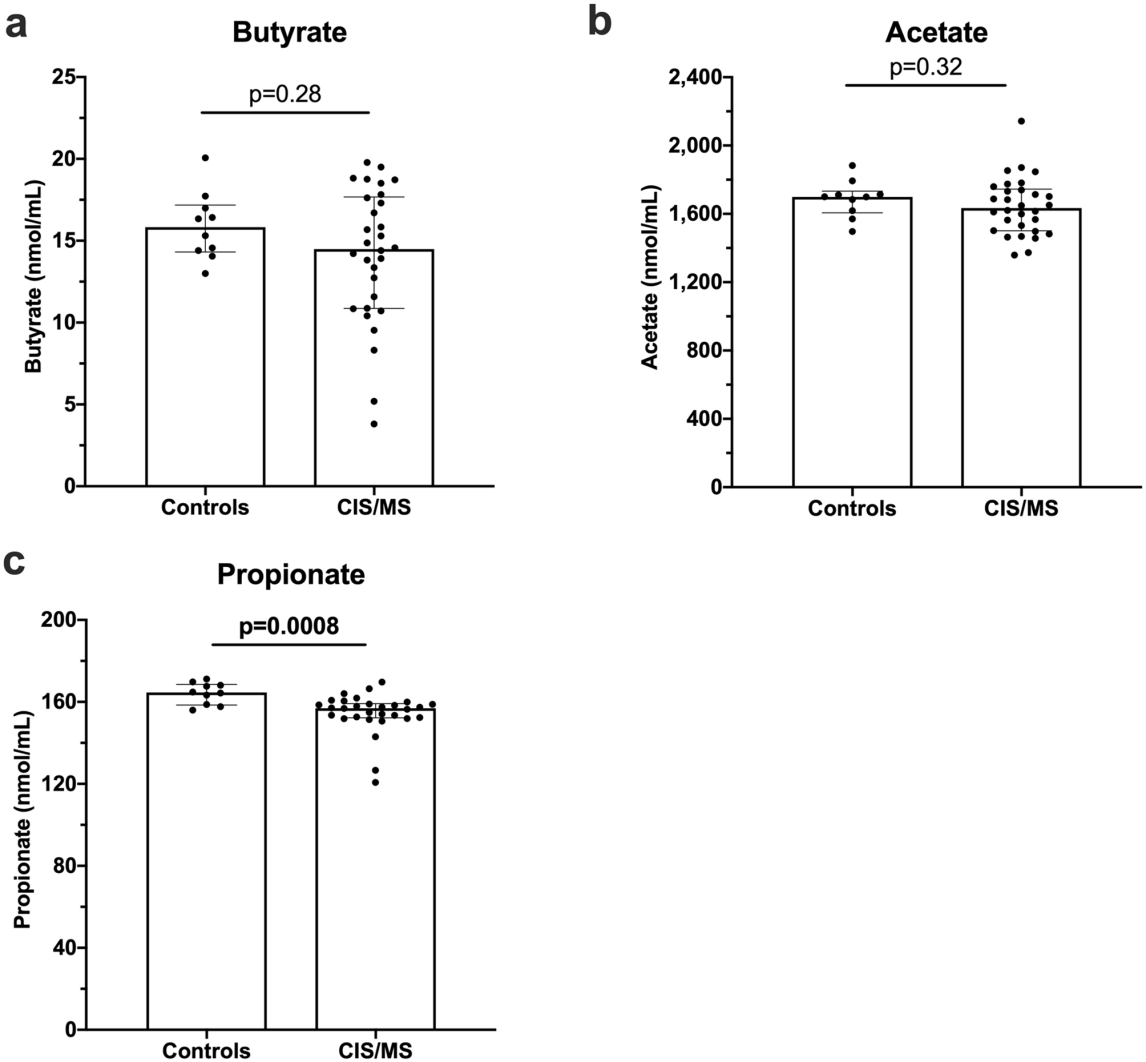
SCFA levels in serum from controls (n = 10), and patients with CIS or MS (CIS/MS; n = 30). **(a)** Butyrate**, (b)** Acetate, and **(c)** Propionate levels. Bars show median and interquartile range. Comparisons were made using non-parametric tests (Mann–Whitney tests for two comparisons).

Serum levels of butyrate, acetate and propionate were assessed in CIS/MS patients and compared to those in healthy controls. A small but significant difference was observed for propionate levels between controls and CIS/MS patients (Fig. 1). There was a subset of CIS/MS patients with relatively low serum butyrate or acetate levels, but the levels in the CIS/MS patient group were not significantly different from controls (Fig. 1). There were no associations between levels of any SCFA and the time since their MRI when presenting with CIS or MS (n = 30, data not shown). Serum levels of SCFAs from the CIS patients did not predict the time from their diagnosis with CIS and their conversion to MS by appearance of new lesions on their MRI consistent with conversion to MS (n = 16, data not shown).

### Association of SCFAs with the prevalence and function of peripheral blood T- and B-cell subsets from CIS/MS patients

This laboratory has analysed both the prevalence and function of circulating B- and T-cells of therapy-naive patients recently presenting with CIS or MS^26–29^. Due to the variable numbers of samples that had been available at the time of each assessment, there was some minor variation in the sample numbers between analyses (as indicated).

#### Serum SCFA levels and the frequency of T-cell subsets and their function

In the blood from 20 patients with CIS and 5 patients with MS, this laboratory has measured the prevalence of CD4^+^ T-cell subsets^26^. PBMCs from 11 of these 25 patients were cultured with phorbol myristate acetate and ionomycin for 4 h in the presence of Brefeldin A, and the intracellular production of the cytokines IL-17, IFNγ and IL-10 measured in CD4^+^ T-cells^26^.

**Figure 2:**
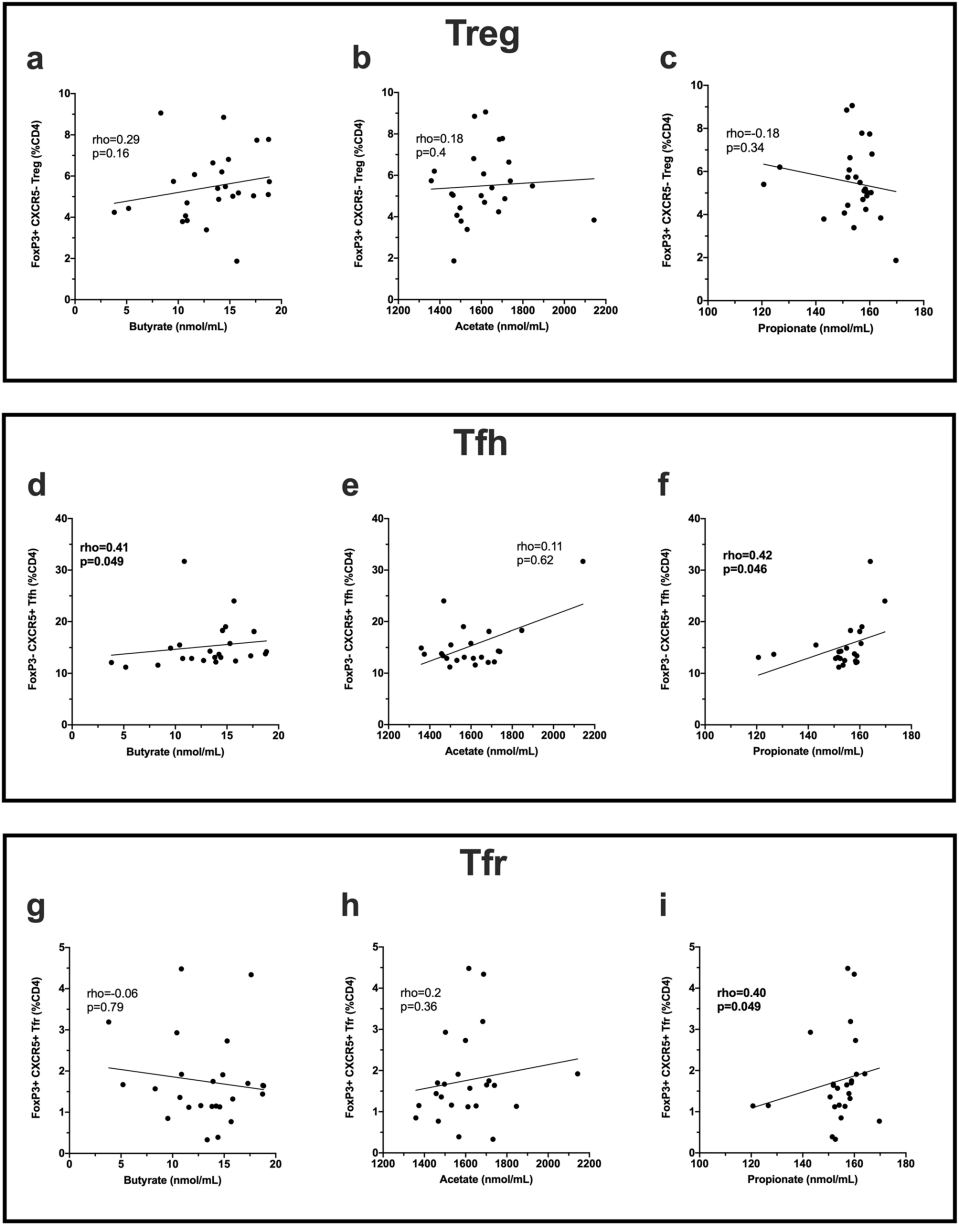
Correlations between serum SCFA levels in patients with CIS/MS and CD4^+^ T cell subsets. Top panel shows T regulatory cells (Treg; CD4^+^Foxp3^+^CXCR5^−^; n = 25), middle panel shows T follicular helper cells (Tfh; CD4^+^CXCR5^+^; n = 23), bottom panel shows T follicular regulatory cells (Tfr; CD4^+^Foxp3^+^CXCR5 + ; n = 25), and their correlation with **(a,d,g)** butyrate, **(b,e,h)** acetate and **(c,f,i)** propionate levels in serum. Statistical comparisons were made with nonparametric Spearman’s correlation; rho values and p values are shown. Linear regression line is shown for illustrative purposes only. Statistically significant p values are shown in bolded font.

There was no correlation between the levels of serum SCFAs and the prevalence of CD3^+^ cells, CD4^+^ cells, CD8^+^ cells measured as a percentage of PBMC (data not shown). The frequencies of FoxP3^+^CXCR5^−^ T regulatory cells (Tregs; %CD4^+^ T-cells) were not correlated with serum SCFA levels (Fig. 2a–c). In contrast, the frequencies of T follicular helper cells (Tfh) (CD4^+^CXCR5^+^) correlated positively with both serum butyrate and serum propionate levels (Fig. 2d–f).

FoxP3^+^CXCR5^+^ T follicular regulatory cells (Tfr; %CD4^+^ T-cells) were also examined: Tfr counteract the functions of Tfh by downregulating the production of effector cytokines such as IL-4, IFNγ and IL-21 that are essential for B-cell activation and class switch recombination^30–32^. In our CIS/MS cohort (n = 25), the frequency of Tfr correlated with levels of propionate, but not other SCFAs in serum (Fig. 2g–i). In addition, propionate levels significantly negatively correlated with the relative ratio of Treg:Tfr (rho -0.43; p = 0.03). For butyrate and acetate, there was no significant correlation with the Treg:Tfr ratio (rho = 0.08, p = 0.7 and rho = 0.02, p = 0.9, respectively). There was no correlation between serum acetate, propionate or butyrate levels and the ratio of Tfh:Tfr (n = 23; rho = − 0.07, p = 0.74; rho = − 0.1, p = 0.64; rho = 0.05, p = 0.82, respectively).

No correlation was found between serum SCFA levels and Th1 or Th17 cell frequencies (%CD4^+^ T-cells) (n = 25 CIS/MS) or expression of IL-10, IFNγ or IL-17 in total CD4^+^ T-cells (n = 11 CIS/MS) (data not shown).

In summary, a relationship was demonstrated between serum levels of some SCFAs and the frequencies of circulating follicular T-cells (Tfh or Tfr cells) but not the frequency or cytokine production of other subpopulations of CD4^+^ T-cells.

#### Serum SCFA levels and the frequency of B-cell subsets and their function

In the blood from 21 patients with CIS/MS, the frequencies of eight CD19/CD20^+^ B-cell subsets were previously measured^29^. We have also cultured PBMCs from these patients for 18 h and measured the intracellular production of the prototypical pro- and anti-inflammatory cytokines TNF and IL-10 in B-cell subsets, both in the absence and presence of R848, a polyclonal stimulator of B-cells through the TLR7 receptor^29^.

**Figure 3:**
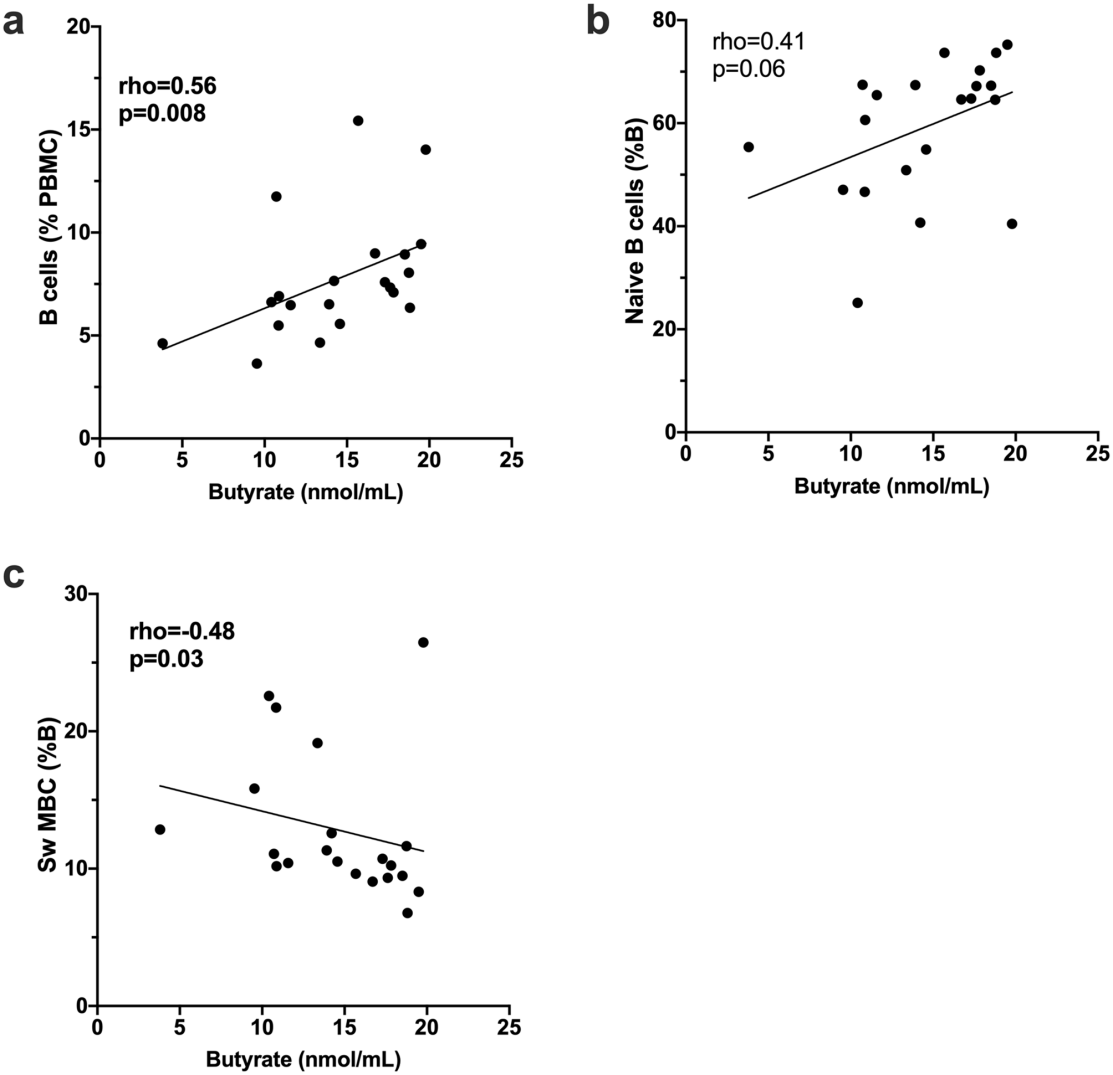
Correlations between serum butyrate levels and B cell frequencies in **(a)** total B cells (% PBMC), **(b)** naive B cells (% B cells) and **(c)** class switched memory B cells (Sw-MBC; % B cells). Data are from PBMC isolated from 21 CIS/MS patients. Correlation was tested using nonparametric Spearman correlation; rho and p values are shown. Line of regression is shown for illustrative purposes only. Statistically significant p values are shown in bolded font.

The frequencies of total B-cells (% PBMC) correlated positively with serum levels of butyrate for CIS/MS patients (n = 21; Fig. 3a). Similarly, naive B-cell frequencies (% B-cells) appeared positively correlated with serum butyrate but this was not statistically significant (p = 0.06; Fig. 3b). In contrast, frequencies of class-switched MBCs (%B-cells) were significantly negatively correlated with butyrate levels (p = 0.03; Fig. 3c). No other correlations were observed between serum SCFA levels and frequencies of B-cell subsets (naive, transitional, IgM^hi^ MZ-like B-cells, IgM^lo^ MZ-like B-cells, IgM-only MBC, IgM^+^ double negative (IgD^−^CD27^−^; DN), IgM^−^ DN, and plasmablasts as %B-cells) (data not shown).

**Figure 4:**
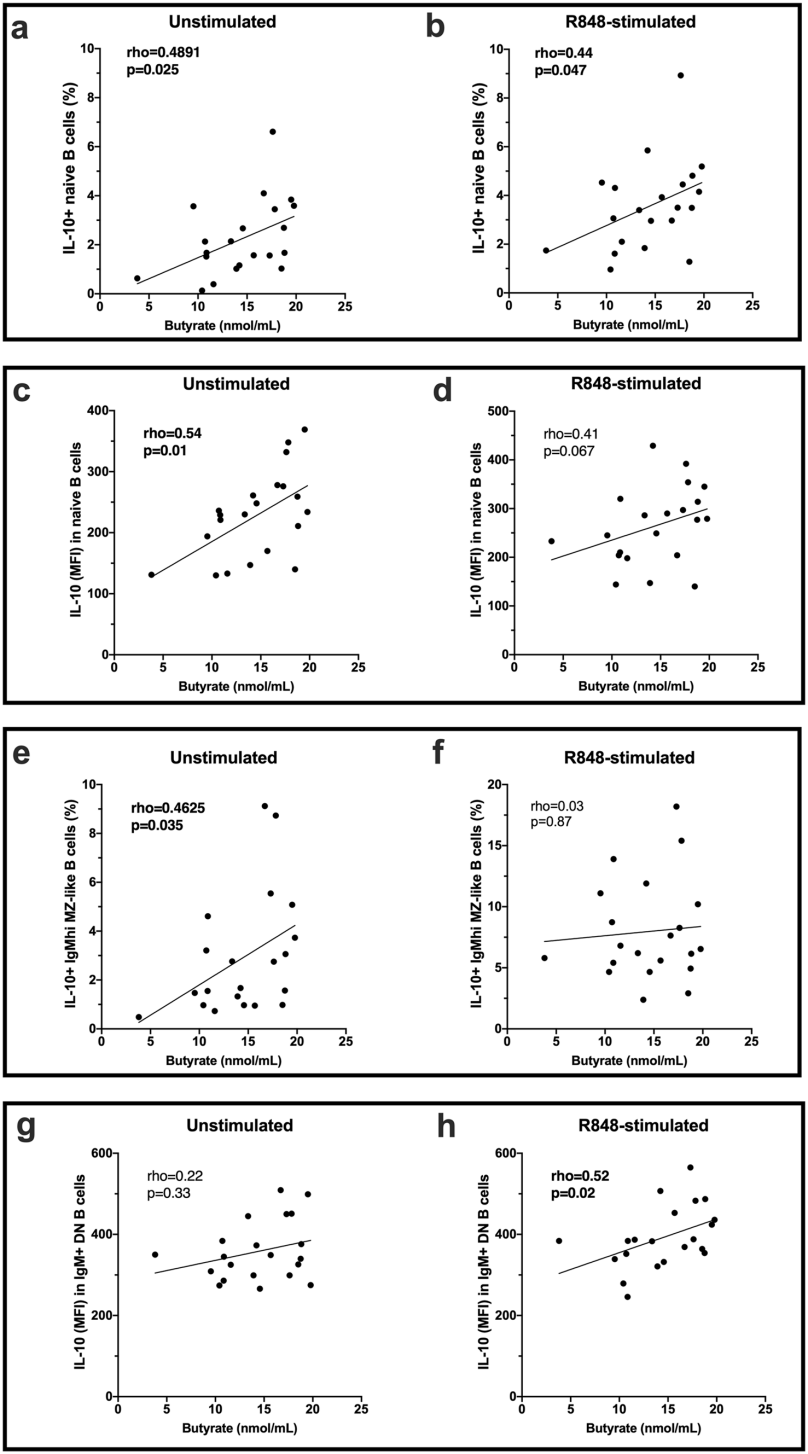
Correlations between serum butyrate levels and IL-10-producing B cells after PBMC culture for 18 h (n = 21). Cells were cultured with or without Resiquimod (R848) as indicated. **(a,b)** The proportion of unstimulated or R848-stimulated naive B cells expressing IL-10 (%) and **(c,d)** the expression (MFI) of IL-10 in unstimulated or R848-stimulated naive B cells. **(e–f)** The proportion of IgM^hi^ MZ-like B cells expressing IL-10 in unstimulated or R848-stimulated cells. **(g–h)** The expression of IL-10 (MFI) in unstimulated or R848-stimulated IgM^+^ DN B cells. Correlation was measured using nonparametric Spearman’s rho; statistically significant values are shown in bolded font. Line of regression is shown for illustrative purposes only.

When PBMCs were cultured for 18 h in the absence of exogenous stimulation and the frequency of IL-10-expressing B-cells compared with levels of SCFAs, the percentage of both naïve and IgM^hi^ MZ-like B-cells that expressed IL-10 correlated positively with the concentration of butyrate in serum samples from patients with CIS/MS (Fig. 4a,e). There was no significant correlation of SCFAs with IL-10 production by the other B-cell subsets. The median fluorescence intensity (MFI) for IL-10 in unstimulated naive B-cells but not other B-cell subsets, also correlated with serum butyrate levels (Fig. 4c,g). In R848-stimulated B-cells, the frequency of naive B-cells, but not IgM^hi^ MZ-like B-cells, expressing IL-10 correlated with serum butyrate levels (Fig. 4b,f). Upon R848-stimulation, the MFI of IL-10 in IgM^+^ DN B-cells correlated significantly with serum butyrate levels (Fig. 4h) and was close to significant in naïve B-cells (Fig. 4d).

**Figure 5:**
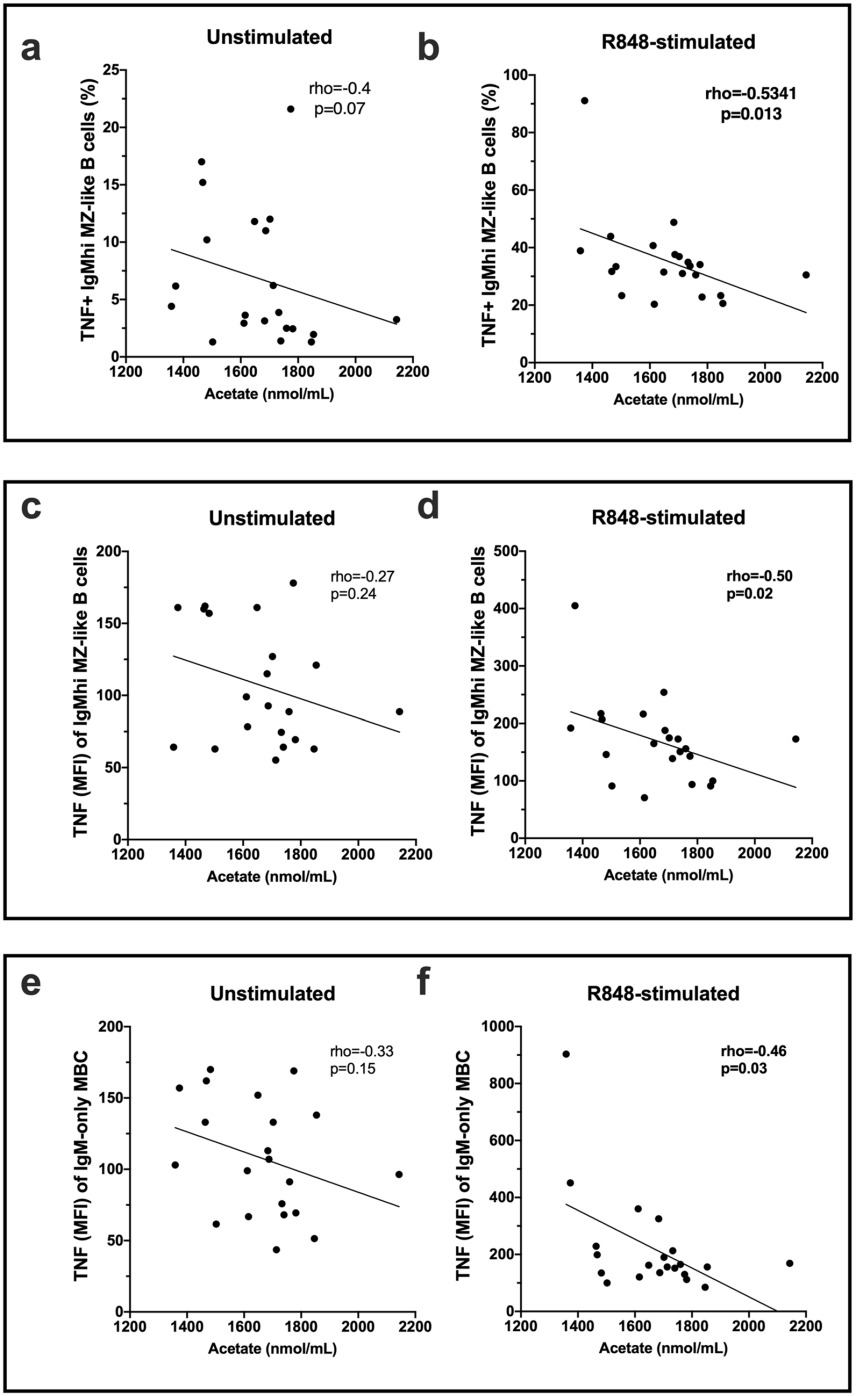
Correlations between serum acetate levels and TNF-producing B cells after PBMC culture for 18 h (n = 21). Cells were cultured with or without Resiquimod (R848) as indicated. **(a,b)** The proportion of unstimulated or R848-stimulated IgM^hi^ MZ-like B cells expressing TNF (%) and **(c,d)** the expression (MFI) of TNF in unstimulated or R848-stimulated IgM^hi^ MZ-like B cells. **(e,f)** The expression of TNF (MFI) in IgM-only memory B cells in unstimulated or R848-stimulated cells. Correlation was measured using nonparametric Spearman’s rho; statistically significant values are shown in bolded font. Line of regression is shown for illustrative purposes only.

When serum SCFA levels were tested for association with TNF production by B-cell subsets, acetate negatively correlated with TNF^+^ IgM^hi^ MZ-like B-cell frequencies in R848-stimulated but not unstimulated cells (Fig. 5a,b). In addition, the expression of TNF (MFI) by both the IgM^hi^ MZ-like B-cells and IgM-only MBC correlated negatively with serum acetate levels for R848-stimulated samples (Fig. 5c–f).

In summary, serum butyrate levels significantly associated with IL-10 expression in some unstimulated and R848-stimulated IgM^+^ B cell subsets, and serum acetate was negatively associated with the capacity of some stimulated IgM^+^ B cell subsets to produce TNF.

### Associations of serum SCFA levels with tryptophan and arginine catabolic enzyme mRNA expression in PBMC

**Figure 6:**
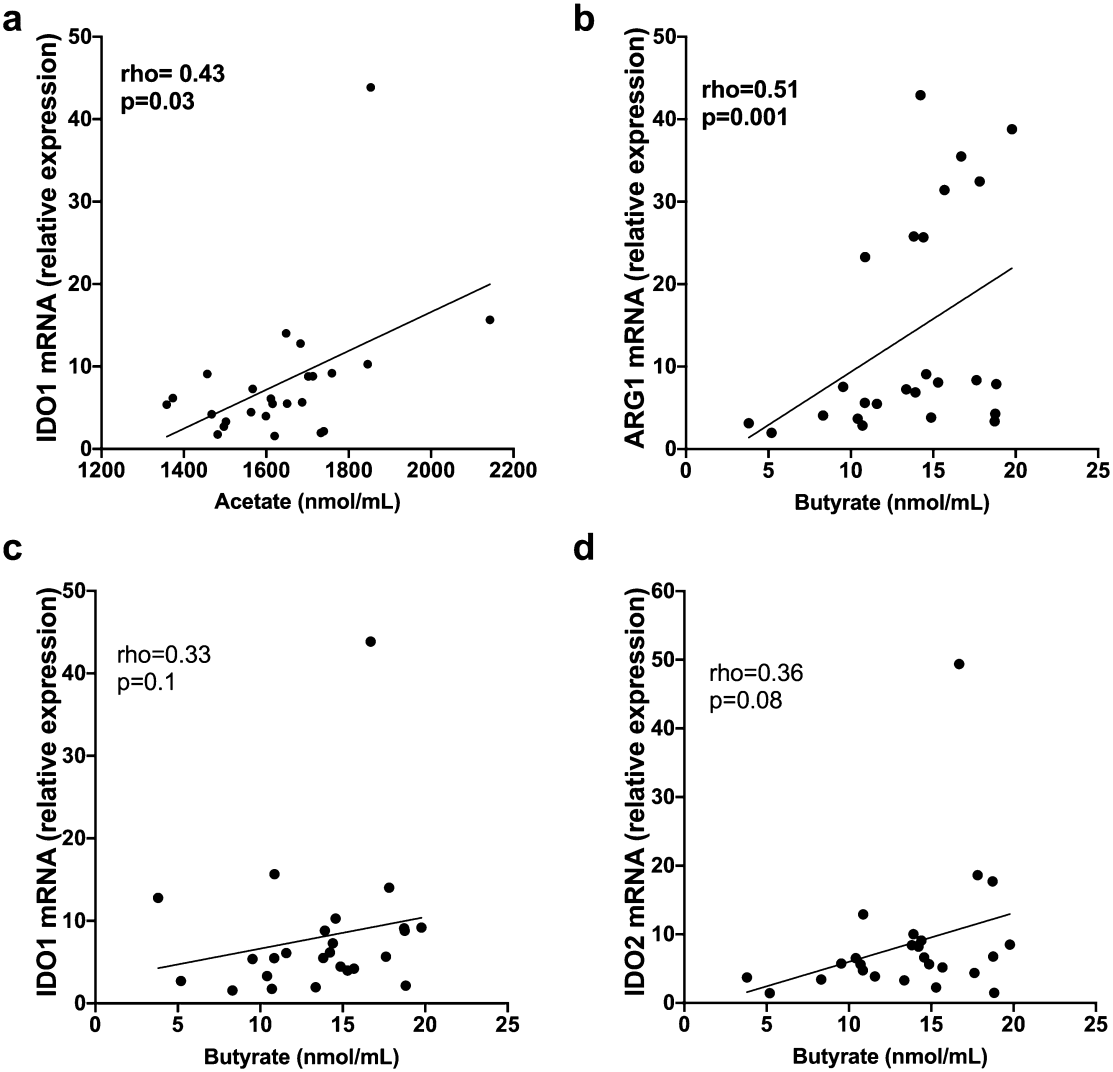
Correlations between serum levels of SCFA in patients with CIS/MS and PBMC mRNA for IDO and ARG (n = 25). **(a)** Correlation of IDO1 mRNA with acetate; **(b)** Correlation of ARG1 with butyrate; **(c)** Correlation of IDO1 mRNA with butyrate; and **(d)** Correlation of IDO2 mRNA with butyrate. Correlation was measured using nonparametric Spearman’s rho; statistically significant values are shown in bolded font. Line of regression is shown for illustrative purposes only.

We have previously published that levels of mRNA for indoleamine 2,3-dioxygenase (IDO)1/2 and arginase (ARG)1/2 are increased in PBMCs from CIS/MS patients^33^ and might provide a sustained homeostatic mechanism to control MS-associated inflammation. Expression of these enzymes in PBMCs from 25 CIS/MS patients was compared with serum SCFA levels, since butyrate has been reported to increase IDO1 expression in dendritic cells in culture and their ability to promote Treg differentiation^34^. IDO1 mRNA levels correlated positively with serum acetate levels, and ARG1 levels with serum butyrate (Fig. 6a-d). However, IDO1 and IDO2 mRNA levels did not correlate significantly with butyrate levels (p = 0.1 and p = 0.08, respectively).

### Associations of serum SCFA levels with other disease biomarkers in serum from CIS or MS patients

Other disease biomarkers potentially involved in MS development and whose levels may associate with SCFA levels were examined in aliquots of serum from patients with CIS/MS.

**Figure 7:**
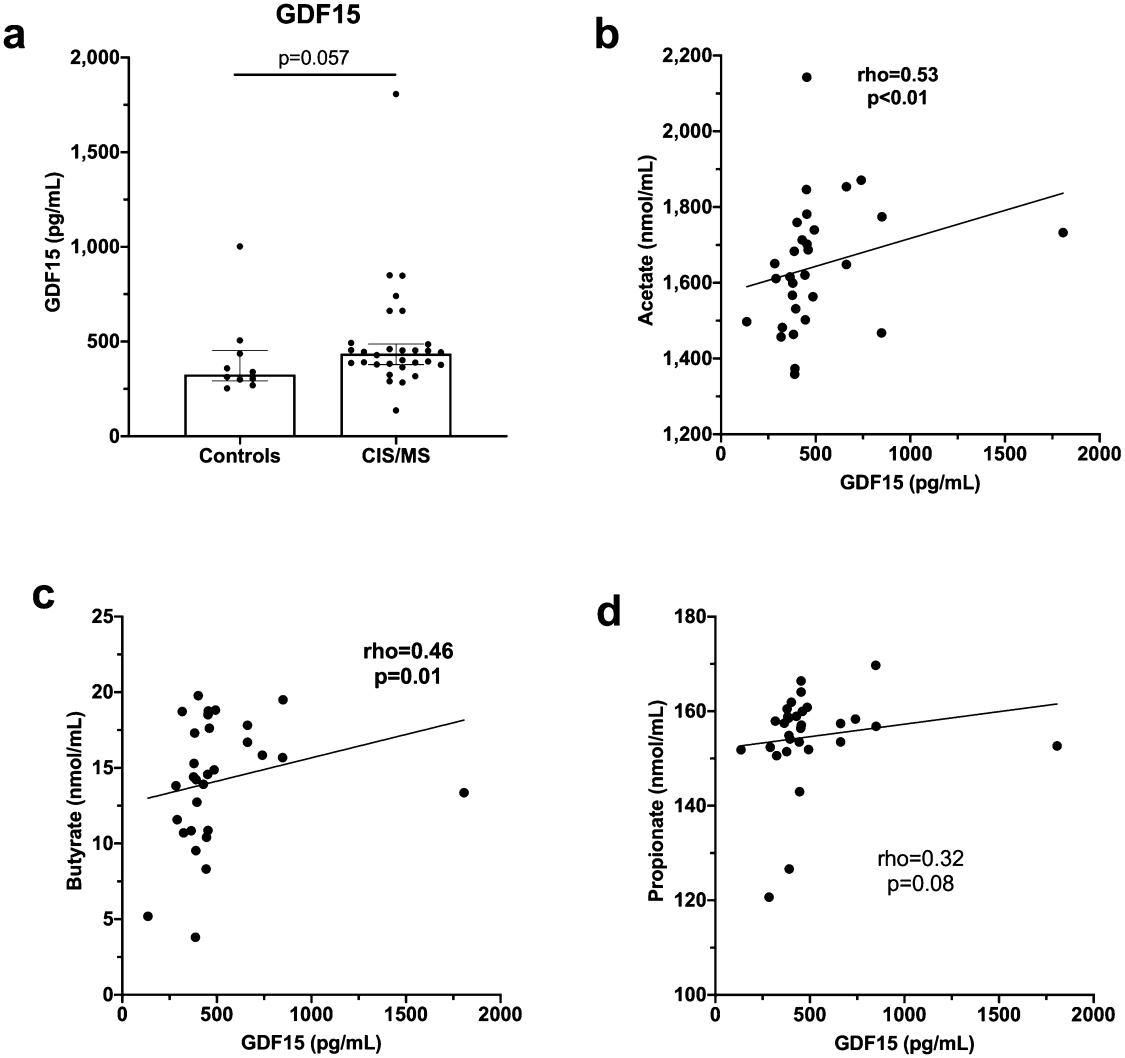
GDF15 levels in sera and correlations with SCFA levels**. (a)** GDF15 levels in serum from controls (n = 10) compared with those from patients with CIS/MS (n = 30). Correlation between GDF15 in serum from patients with CIS/MS and serum levels of **(b)** acetate**, (c)** butyrate, and **(d)** propionate. Correlation was analysed using nonparametric Spearman’s rho; p values < 0.05 were considered statistically significant and are shown in bolded font. Linear regression lines are shown for illustrative purposes only.

Serum levels of GDF15 have been reported to be increased in MS patients^24^ and were also higher in patients with CIS/MS (n = 30) in our study although the difference from healthy controls (n = 10) was not statistically different (p = 0.057) (Fig. 7a). Serum levels of GDF15 positively correlated with serum levels of both acetate and butyrate in samples from the CIS/MS patients. A similar trend toward correlation between propionate and GDF15 was not significant (p = 0.08) (Fig. 7b–d).

Given that associations between the frequencies of class-switched MBC and serum butyrate were detected (Fig. 3c) and higher IgG_3_ levels associate with CIS to MS conversion^21^, associations were investigated between levels of SCFA and different immunoglobulins. No correlation was detected between SCFA concentrations and levels of IgG, IgM, IgA, or IgG_2-4_, or their proportions as a percentage of total IgG (n = 28 CIS/MS) (data not shown). In addition, serum B-cell activating factor (BAFF) which is associated with B-cell survival and is increased in serum from patients with MS^35^, was investigated, particularly as we detected increased frequencies of B-cells (%PBMC) in association with serum butyrate (Fig. 3a). However, There was no significant correlation between levels of SCFAs and serum BAFF (n = 20 CIS) although a trend toward a negative correlation between BAFF and acetate was observed (rho = − 0.39, p = 0.09) (data not shown).

There was no significant correlation between serum levels of SCFAs and 25(OH)D (n = 28 CIS/MS)(data not shown), the latter being a molecule frequently implicated in MS development if levels are deficient^23^.

## Discussion

In this study of SCFAs in the serum of CIS/MS patients, propionate levels were significantly lower than those measured in the serum of healthy controls. Furthermore, several CIS/MS patients had butyrate and acetate levels that were below the range of levels measured in the serum from the healthy controls. The spread of levels of SCFAs may reflect the heterogeneity of the CIS/MS cohort but we were not able to associate lower levels with a clinical outcome. We aimed to determine whether associations of SCFAs with cells and mediators in the same blood sample taken soon after disease presentation may give clues to putative gut microbiota/SCFA involvement in MS pathogenesis, particularly by regulation of immunometabolic processes. Many correlations were detected between levels of SCFAs and circulating immune cells and their products, that suggested an unconfirmed potential of SCFAs to support T follicular cells and IL-10-expressing, less-differentiated B-cells, and to inhibit B-cell class switching and B-cell TNF expression. Overall, our results reflect a broad association of different SCFAs with immune cells that may contribute to regulation of both adaptive (T- and B-cell) and innate (myeloid cell) immune responses.

A significant positive association was detected of propionate with CXCR5-expressing Tfh and Tfr. These cells are associated with germinal centre reactions in secondary lymphoid organs as CXCR5 expression enables cell migration into germinal centres^36,37^. Propionate levels significantly positively correlated with the frequency of Tfr but not Treg cells; this result suggests the effects of decreased propionate in patients with CIS/MS may be more important for circulating Tfr than Treg. In MS, fewer circulating functional Tfr have been implicated in the emergence of autoreactive B-cells, breakdown of self-tolerance and the emergence of ectopic germinal centre-like structures within the meninges^38^. In mouse models, butyrate and propionate supplementation have induced maturation of Tfr from Tregs via histone acetylation in Tfr marker genes^39^. Therefore, lower propionate levels in the serum from CIS/MS patients could result in decreased regulation of germinal centre activity.

Lower levels of propionate and/or butyrate in the serum of MS patients^10,11^ have been previously reported to associate with lower frequencies of Treg. In addition, Treg differentiation in the presence of these SCFAs has been observed in culture^10,11,40^. However, in these previous studies, Treg cells were defined as CD4^+^CD25^+^FoxP3^+^ but staining for CXCR5 was not performed. Thus, Treg and Tfr populations were not separately analysed; we have published that Tfr represent approximately 24% of total CD4^+^CD25^+^Foxp3^+^ cells in both healthy controls and CIS patients^26^. In our study, Treg cells were defined as CD4^+^FoxP3^+^CXCR5^−^ and no associations with SCFAs were demonstrated in the CIS/MS cohort. Tfr originate from CXCR5^−^FoxP3^+^ thymic-derived Treg through dendritic cell priming (reviewed in^41^); in vitro studies are required to determine how propionate may regulate the differentiation of human Tfr from Treg.

The success of anti-CD20 therapies^42^ has highlighted the importance of B-cells in the immunopathogenesis of MS. Associations between B-cells and SCFAs were frequent. Levels of serum butyrate positively correlated with the frequency of total B-cells (as% PBMCs) and naive B-cells (as% B-cells). Butyrate levels also negatively associated with the proportion of class-switched MBC. These results suggest that butyrate might affect B-cell differentiation in humans, a proposition that is supported by a report that butyrate and propionate fed to mice can reduce B-cell class-switching and plasma cell differentiation^43^. This effect of butyrate may be complemented by propionate stimulating the number of Tfr. Butyrate levels also associated positively with both endogenous and TLR7-induced IL-10 production by several IgM^+^ B-cells subsets. Lower levels of serum acetate associated with increased production of TNF by B-cell subsets. Thus, lower butyrate and acetate levels may induce B-cell subsets to be more inflammatory with decreased IL-10 and increased TNF production; outcomes supported by the ability of SCFAs to epigenetically edit the transcription of cytokine genes^18,43,44^. An indirect effect of butyrate on B-cells may involve the microbial metabolism of tryptophan, causing the activation of the aryl-hydrocarbon receptor on B-cells and promoting their differentiation to B-cells with regulatory potential^45^.

Serum levels of acetate also positively correlated with PBMC mRNA levels of the tryptophan catabolic enzyme, IDO-1. Butyrate positively correlated with ARG-1 mRNA levels, an arginine catabolic enzyme principally expressed by macrophages. Both enzymes reduce amino acids for activation and expansion of immune cells and allow production of downstream immunomodulatory metabolites^46^. Levels of serum GDF15 correlated positively with serum butyrate and acetate levels and a similar trend was observed for propionate (p = 0.08). The involvement of GDF15 in MS pathogenesis requires further study. However, GDF15 is neurotrophic and in a model of CNS injury, transgenic overexpression of GDF15 benefited injury resolution by modifying the peripheral immune response^47^. This study suggests that levels of serum GDF15 may be inter-related with SCFA-producing gut microbiota. Vitamin D determined principally by exposure of skin to UV radiation^48^ may alter the gut microbiome and in turn, SCFA levels. However, serum levels of 25(OH)D showed no correlation with SCFAs (data not shown).

The strength of our report is that to our knowledge, such a thorough analysis of associations of SCFA levels with immune cells and serum disease biomarkers has not previously been performed. In previous studies of serum SCFA associations with MS, patients generally lived in the northern hemisphere and at higher latitudes. This study was performed in Perth, Australia (32^o^S), where the local diet of the patients may have differed for seasonal, cultural and geographical reasons. It is important that in our study, like that of Duscha and colleagues in Germany^11^, lower levels of propionate were detected in the serum of CIS/MS patients. As MS is likely initiated by peripherally-activated, autoreactive lymphocytes that can then cross the blood–brain barrier (reviewed in^49^), our study is relevant as serum SCFAs were analysed against circulating immune cells and mediators that subsequently may, upon entry into the central nervous system, be potential drivers of myelin-degrading cells.

The limitations of the study include our inability to determine whether the associations reported are also relevant to a control population. There was power to examine SCFA associations with the frequencies of cells and mediators only in samples from the CIS/MS patients. Further associations particularly for T-cell subsets may be detected with larger numbers of patients. Our analyses could not determine the direction of the communication between the circulating immune cells and biomarkers, and the gut microbiota. The communication is most likely bidirectional, whereby SCFAs affect not only immune processes but also, immune cells and their mediators regulate the gut microbiota. For example, it has recently been reported that arginase can alter the microbiome^50^. In addition, the gut microbiome can be considered a hub for integrating signals from the immune and other body systems^51^. Our correlative studies could not determine whether there may be redundant, additive or synergistic actions of the SCFAs on immune cells. Also, the site of interaction of SCFAs with developing immune cells that allow associations to be detected in the blood, could not be determined and may reflect initiation at the gut wall, in the bone marrow, or in secondary lymphoid organs. However, the associations observed in this study align with findings from experimental studies supporting a potential ability of SCFAs to regulate immune cells and their function in MS. The clinical outcomes investigated were very limited so any prognostic value of reduced SCFA concentrations would be premature.

To conclude, the measurement of SCFAs in the serum of patients with CIS/MS provided a window to dissect potential mechanisms of gut-brain communication that may be associated with MS development, and to pinpoint the immune cells and mediators that may be intermediates in that pathway. Although only associative, this study adds supportive data to emerging proposals that changes in diets for MS patients could increase the production of beneficial SCFAs via changes to the microbiome, or MS patients be supplemented with particular SCFAs or pre/probiotics to improve clinical outcomes^11,52,53^. In our study, SCFA levels were associated principally with processes reflecting the function of germinal centres and IL-10-producing B-cells, rather than Treg cells. In serum from our CIS/MS cohort, propionate was significantly reduced compared with controls and highlights the functions of propionate on cells controlling germinal centre reactions (Tfh and Tfr) as a potentially important process in MS pathogenesis. Further studies of SCFAs and blood cells in vitro, and SCFA supplementation studies in vivo, will help to further define the mechanisms of action of SCFAs, and their possible association with factors determining the development and progression of MS.

## Methods

### Donors of PBMC and serum

Samples from patients with clinically isolated syndrome (CIS), the earliest form of MS (n = 20) were taken from the PhoCIS trial^54^. Serum and cells from their baseline blood donation were analysed, which was taken within 120 days of their diagnosis of CIS by neurological history, examination, appropriate laboratory investigations and magnetic resonance imaging (MRI). All were non-smokers and of Caucasian ethnicity. Patients defined as MS (n = 10) were typically those with an acute clinical episode and were recruited based on a recent MRI showing demyelinating disease consistent with MS^21^. From their MRI result, they were ineligible for recruitment for the PhoCIS trial. All CIS and MS patients were MS disease modifying therapy naïve and had not received steroids for > 30 days; they all had minimal disability due to the early nature of their disease (Expanded Disability Status Scale of < 3)^54^. In this study to increase the power of our analyses, the CIS and MS patients were considered as a single CIS/MS group (n = 30). Ten control age- and sex-matched individuals with no history of autoimmune disease were also recruited for serum donation. Details of the controls and CIS/MS patients are shown in Table 1. Donors of PBMC and serum were not fasting at time of venesection.

**Table 1:** Demographic details of controls and patients with clinically isolated syndrome (CIS) or multiple sclerosis (MS).

	Controls (n = 10)	CIS/MS (n = 30)	p-value
Age (years; median, (interquartile range))	35.4 (26.8–42.8)	34.5 (29.8–45.2)	0.4
Sex (female; n (%))	8 (80%)	19 (63.3%)	0.29

Statistical comparisons were made using a Mann–Whitney test for age and Fisher’s exact test for sex.

The study of the controls and CIS patients was approved by the Bellberry Human Research Ethics Committee (2014–02-082) and endorsed by the Human Research Ethics Office of the University of Western Australia (RA/4/1/6796). The study of the MS patients was reviewed and approved by the Sir Charles Gairdner Hospital Human Research Ethics committee (2006–073). All participants provided their written informed consent to participate. All experiments were performed in accordance with relevant named guidelines and regulations.

### Serum and PBMC collection

Peripheral venous blood was collected into sodium heparin and SST vacutainers (BD Biosciences, North Ryde, Australia) to isolate peripheral blood mononuclear cells (PBMC) and serum, respectively. Serum was following centrifugation at 800 × *g* for 10 min at room temperature and stored at -80 ºC until analyses. PBMC were separated from whole blood using a Lymphoprep (Axis-shield, Dundee, UK) gradient and cryopreserved as previously described^28^.

### SCFA measurement

As previously described^55^, serum samples were spiked with ^13^C-sodium acetate, ^13^C-sodium butyrate and ^13^C-sodium propionate (all from Sigma) as internal standards, acidified and homogenised in isopropanol. Following centrifugation, the supernatant was collected and injected into an Agilent HP 6890 Series GC System, equipped with an Agilent 5973 Network Mass Selective Detector in splitless mode. Samples were separated on a DB Waxeter column (30 m × 0.25 mm × 0.25 μm), using a helium carrier gas at a flow rate of 1.0 mL/min. Identities and retention times of the SCFA were established using the volatile-free acid mix and were manually integrated using the MSD ChemStation (version D.03.00.611). Concentrations were determined using the internal standard references and calculated as nanomoles per microlitre of serum.

### Assessment of T-cell frequencies and cytokine production by flow cytometry

As previously described and graphically shown^26^, T-cells (CD3^+^) were identified using multicolour flow cytometry, including T regulatory cells (Tregs; CD4^+^FoxP3^+^CXCR5^−^), T follicular regulatory cells (Tfr; CD4^+^FoxP3^+^CXCR5^+^), T follicular helper cells (Tfh; CD4^+^FoxP3^−^CXCR5^+^), Th1 cells (CD4^+^CXCR3^+^CCR6^−^) and Th17 cells (CD4^+^CXCR3^−^CCR6^+^). The expression of intracellular cytokines IL-10, IFNγ and IL-17 in CD4^+^ cells was examined in a subset of individuals with CIS (n = 11). Cytokines were examined in total PBMC using flow cytometry following a 4 h stimulation with phorbol 12-myristate 13-acetate and ionomycin in the presence of brefeldin A, as previously described^26^.

### Assessment of B-cell frequencies and cytokine production by flow cytometry

B-cells were identified as CD19/CD20^+^ cells and subsets defined as previously described and illustrated^29,56,57^ including transitional, naive marginal-zone (MZ)-like (both the IgM^hi^ and IgM^lo^ subpopulations), IgM-only memory B-cells (MBC), class-switched MBC (Sw MBC), and IgM^+^ or IgM^−^ double negative B-cells. Intracellular IL-10 and TNF expression was examined in B-cell subsets cultured within PBMC for 18 h, with or without Resiquimod (R848), a TLR7/8 ligand, and in the presence of GolgiPlug, using flow cytometry as previously described^29^. Cytokine expression by B-cell subsets was measured both as the proportion of positive cells (percentage of total B-cells) and the amount of cytokine expressed per cell (median fluorescence intensity (MFI)).

### Measurement of IDO1/2 and ARG1/2 mRNA

As previously published^33^, PBMCs were lysed, RNA was extracted and cDNA synthesis performed by standard techniques. Levels of mRNA for indoleamine 2,3-dioxygenase (IDO) 1 and IDO2, and arginase (ARG) 1 and ARG2 were determined by RT-PCR and relative gene expression determined.

### Measurement of GDF15, BAFF, immunoglobulins and 25(OH)D

GDF15 and BAFF were measured using ELISA kits according to the manufacturer’s instructions (R&D Systems, Minneapolis, MN, USA). Total IgG, IgG_2–4_, IgM, and IgA were measured as previously described, using bead-based commercial immunoassays^21^. 25(OH)D was measured in serum by LC–MS/MS as previously published^54^.

### Statistical analyses

The data were not normally distributed according to Shapiro–Wilk normality tests, and therefore nonparametric data analyses were performed. Associations between continuous variables in patients with CIS or MS were compared in samples using Spearman rank-order correlation. Categorical comparisons were made using Fisher’s exact test. Comparisons of continuous outcomes between controls and those with CIS or MS were made using Mann–Whitney tests. P-values < 0.05 were considered statistically significant. Analyses were performed using SPSS (IBM software v25, Armonk, USA) and Prism software (v8.2.0, GraphPad, San Diego, USA). In figures showing correlation of continuous variables, statistics from Spearman correlation are shown. A line of linear regression has been inserted into figures for illustrative purposes, but no linear regression statistics were included at any point in the analyses due to the nonparametric distribution of the dataset.

## Data Availability

The datasets analysed during the current study are available from the corresponding author on reasonable request.

## Abbreviations

ARG: Arginase
BAFF: B-cell activating factor
CIS: Clinically isolated syndrome
DN: Double negative (IgD^−^CD27^−^)
GDF15: Growth differentiation factor 15
IDO: Indoleamine 2,3-dioxygenase
MBC: Memory B-cells
MFI: Median fluorescence intensity
MRI: Magnetic resonance imaging
MS: Multiple sclerosis
MZ: Marginal zone
SCFAs: Short-chain fatty acids
SD: Standard deviation
Sw MBC: Class-switched memory B-cells
Tfh: T follicular helper
Tfr: T follicular regulatory
Treg: T regulatory
25(OH)D: 25-Hydroxy vitamin D
